# Study of the Material Removal Mechanism and Surface Damage in Laser-Assisted Milling of CF/PEEK

**DOI:** 10.3390/ma18040791

**Published:** 2025-02-11

**Authors:** Qijia Wang, Minghai Wang, Li Fu, Kang Xiao, Xuezhi Wang

**Affiliations:** 1 School of Mechatronics Engineering, Shenyang Aerospace University, Shenyang 110136, China; wangminghai2008@163.com (M.W.); xk13841273893@163.com (K.X.); wangxuezhineu@126.com (X.W.); 2 Key Laboratory of Rapid Development & Manufacturing Technology for Aircraft, Shenyang Aerospace University, Ministry of Education, Shenyang 110136, China; 3 Key Laboratory of Fundamental Science for National Defense of Aeronautical Digital Manufacturing Process, Shenyang Aerospace University, Shenyang 110136, China; 4 School of Automation, Shenyang Aerospace University, Shenyang 110136, China; ffulli@163.com

**Keywords:** laser-assisted machining, CF/PEEK composites, surface damage, milling quantity

## Abstract

Carbon-fiber-reinforced polyetheretherketone (CF/PEEK) composites are being increasingly used in aerospace, biomedical, and other industries due to their superior mechanical properties. However, CF/PEEK structural components require secondary processing after curing and molding to meet connection and assembly precision requirements. This process, however, often results in defects such as burrs and pits, which significantly compromise the mechanical performance and assembly quality of the structural components. This study first employed finite element simulations to analyze the laser-assisted milling of CF/PEEK composites, investigating the material removal mechanism under thermal coupling, which was then experimentally validated. Variations in the cutting force, cutting heat, surface damage, and fiber fracture mechanisms during milling were investigated. During laser-assisted milling, the fibers fractured mainly in bending at a cutting angle of 0°, in bending shear at a cutting angle of 45°, in compression at a cutting angle of 90°, and in compression shear at a cutting angle of 135°. The experimental findings were generally consistent with the simulation results. In addition, laser-assisted milling effectively reduced the cutting forces, cutting temperatures, and surface damage compared to conventional milling; laser-assisted milling reduced the cutting forces in the 90° fiber direction by 24.8% (total cutting forces) and 16.3% (feed-cutting forces). The fiber integrity was further increased with increasing spindle speed.

## 1. Introduction

High-performance carbon-fiber-reinforced thermoplastic composites (CFRTPs), composed of an organic polymer matrix and carbon fiber reinforcement, offer advantages such as an excellent toughness, thermoformability, recyclability, weldability, and convenient storage over traditional carbon-fiber-reinforced composites [1,2]. These properties enable widespread applications in the aerospace, precision instrumentation, and defense industries [3]. CF/PEEK, a high-performance CFRTP, contains a thermoplastic PEEK matrix with a glass transition temperature (Tg) of 143 °C. It has an excellent biocompatibility and an excellent resistance to chemicals, heat, and abrasion. In addition, thermoplastic materials have a higher tendency to absorb water than thermoset plastics, which makes water-based cutting methods, such as water jet cutting, less desirable for processing CFRTPs [4]. However, due to the anisotropic and heterogeneous structure of CFRTPs, similar to that of CFRPs, the cutting tool alternately interacts with fibers and the matrix during machining. This alternation generates a complex material removal mechanism, with a significant effect on the quality of machined composite components [5]. Similar to other CFRTPs, CF/PEEK composites are commonly fabricated by welding or secondary machining, including turning, milling, drilling, and grinding [6]. Since CFRTP materials have a low thermal conductivity and tend to soften when processed at high temperatures, methods such as dry processing and laser-assisted processing (LAC) are commonly used [7]. Conventional milling (CM) processes often cause surface defects, including delamination, fiber pull-out, burr formation, microcracks, surface depressions, and micro-cavities [8,9].

To address machining defects in CFRTP milling, extensive research has been conducted on fiber orientation and machining parameters, focusing primarily on the fiber angle, feed rate, and cutting speed [10]. Fiber pullout occurs at a cutting angle of 0°, fiber pullout and surface depression occur at a cutting angle of 45°, and surface microcracks are more common at a cutting angle of 90°. Improper processing parameters are the main cause of surface thermal damage. Excessive cutting temperatures soften the matrix material, exacerbate its separation from carbon fibers, and ultimately result in machining defects such as fiber pullout and surface depressions [8]. Zhang et al. [11] found that fiber orientation significantly influences subsurface damage and its mechanisms, even with identical machining parameters. The surface roughness, subsurface damage, and cutting forces significantly depend on the fiber orientation. Kumar et al. [12] studied the dry milling of CFRPs and found that the surface roughness was the worst at a 135° cutting angle. The best surface roughness was found at a cutting angle of 45°, but there were still defects such as fiber exfoliation, matrix debonding, and fiber breakage. As the cutting speed increases, the surface quality worsens, while the surface roughness rises with a higher feed per tooth. The feed rate is a key factor affecting surface damage during CFRP milling [13]. Oliveira et al. [14] demonstrated that the primary surface defects during CFRP milling include fiber pullout, matrix peeling, and interlayer delamination. Changes in the feed direction cause different machining defects, while tool wear and a high cutting speed worsen the damage. Notably, tool wear has a pronounced effect on surface roughness. Zhu et al. [15] created 2D and 3D finite element models in Abaqus to study the effect of cutting angles (0°, 45°, 90°, and 135°) on the cutting damage and chip formation, and experimental validation was performed. Liu et al. [16] used single fibers to develop a micromechanical model of CFRP laminate damage, highlighting the effect of fiber angles on fracture behavior during machining. V.N. Gaitonde et al. [17] developed a second-order mathematical model based on response surface methodology (RSM) between the cutting conditions (cutting speed and feed) and machining performance (power and specific cutting pressure) to analyze the interaction between the cutting conditions and the machining response of polycrystalline diamond (PCD) tools for machining unreinforced and reinforced PEEK composites. Xu et al. [18] performed a comparative analysis of the machining characteristics of two high-performance thermoplastic matrix composites, polyimide (PI) and polyetheretherketone (PEEK), under various cutting conditions. The machinability of carbon-fiber-reinforced PI and PEEK composites was evaluated based on the drilling force, machining temperature, delamination damage, surface morphology, hole accuracy, and tool wear.

Furthermore, researchers are investigating alternative machining methods to enhance the efficiency, reduce costs, and improve the machining quality [19,20]. Zhang et al. [21] used ultrasound-assisted milling to assess the CF/PEEK machinability and developed a model for characterizing fiber fracture. The machined surface and subsurface characteristics were analyzed, and the feed rate–fiber deformation depth relationship was explored, offering insights into the microscopic fracture of CF/PEEK fibers during ultrasonic milling. Lin et al. [22] introduced a water–air jet-assisted laser machining method for CFRPs. The method uses a water jet to form a layer on the workpiece, with auxiliary gas above to enhance the impact. The experimental and simulation results showed that this method achieved thermally balanced CFRP removal, improving the processing quality. Rao et al. [23] analyzed the effect of laser parameters on the CFRP surface quality, identifying the cutting speed and the laser power as key factors. Qiao et al. [24] used a femtosecond laser for CFRP perforation with a real-time trimming method (RTM). This method eliminates sidewall taper and prevents defects (e.g., grooves, cavities, fiber cross-section shrinkage), enhancing the hole accuracy and quality.

Hybrid processing methods have been explored, with laser-assisted composite processing (LAC) offering notable advantages in reducing thermal and mechanical damage [25]. A pulsed laser–mechanical composite drilling process was employed for the first time, significantly improving the drilling quality of CFRP thick laminates [26]. The method’s advantages, including a reduced thrust, an improved drilling quality, and a longer tool life, were validated by comparative experiments. Li et al. [27] studied the UV laser machining of CFRP laminates, finding that short-pulsed UV lasers reduced heat buildup and the heat-affected zone (HAZ) to about 50 µm. Li et al. [28] introduced a fiber laser–NC milling method for the first time. An investigation of the impact of the processing strategies, cutting parameters, and thermal damage on the surface quality and morphology led to the fabrication of a 10.0 mm thick CFRP plate with a kerf angle under 5.4 µm and no thermal damage.

During the LAC process, the cutting mode primarily shifts to shear cutting, thereby minimizing material damage. However, significant gaps remain in the current research, particularly regarding the understanding of fiber fracture mechanisms and the fundamental processing mechanisms. This study aimed to provide insights into the CF/PEEK laser composite milling model and its influencing factors by introducing a novel simulation approach based on the bare fiber surface post-laser treatment. It sought to uncover the underlying mechanisms of how the cutting speed and fiber orientation influence the machining properties (e.g., milling force, temperature, and surface quality) of CF/PEEK materials, addressing gaps in the existing research. A fiber-bare milling model was developed to examine the impact of LAC technology on fiber fracture in CF/PEEK composites under various machining conditions. Through a systematic analysis of the fiber orientation effects on the machining behavior and thermodynamic properties, this study explored the theoretical and technical foundations for achieving high-quality, low-damage machining. The ultimate goal was to provide innovative theoretical guidance and technical solutions for the efficient, precise machining of CF/PEEK composites, while advancing the development of laser-assisted milling technology in composite processing.

## 2. Laser Composite Machining Milling Modeling

In the comparative analysis of the LAC and CM machining processes, the macroscopic milling model is insufficient for capturing the detailed fiber fracture patterns and subsurface damage morphology. Therefore, a micromachining approach is required to better analyze the milling process. The micro-metamorphic (right-angle) cutting method allows for a more thorough investigation of microscopic chip formation and the mechanisms of fiber fracture and removal during milling. The Abaqus 2021 (6.14) finite element software was used to simulate unidirectional CF/PEEK composite specimens and investigate the micronized right-angle cutting process.

**Figure 1:**
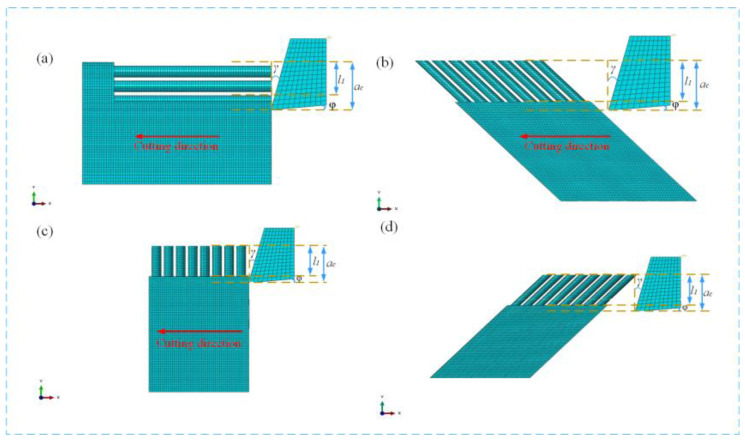
Laser composite cutting model: (**a**) 0°cutting angle, (**b**) 45°cutting angle, (**c**) 90°cutting angle, and (**d**) 135°cutting angle.

The significant thermomechanical property differences between the reinforcing phase and the matrix of CF/PEEK composites cause anisotropy. During laser interaction, the PEEK matrix begins to pyrolyze at about 500 °C (at around 500 °C, the polymer chains of PEEK begin to break, leading to decomposition of the material) [29]. The removal of carbon fibers is carried out at temperatures of approximately 3000 °C or more (in an oxygen atmosphere, the removal of carbon fibers will start at a lower temperature of about 1500 °C to 1700 °C, while in an inert atmosphere, higher temperatures close to 3000 °C are required to start the pyrolysis process significantly) [30]. The laser temperature is kept between the removal thresholds of PEEK and carbon fibers so that the resin is removed and the fibers are retained. Therefore, in the finite element simulation, we simplified the laser action on CF/PEEK by partially removing the PEEK resin matrix and leaving the carbon fibers exposed to achieve the effect after the laser-processing action. The cutting direction in the Abaqus simulation intersects with the axial direction of the carbon fibers (whose cutting direction is the direction of the tool feed) and is rotated clockwise to form an angle *θ*, as shown in Figure 1. The model defines the work piece dimensions as *l × w × h*, and the tool dimensions include the tip radius (*r_e_*), forward angle (*γ*), backward angle (*φ*), depth of cut (*a_c_*), tool width (*h_t_*), fiber bare length (*l_1_*), and cutting speed (*v_c_*) (Table 1). By setting a rigid reference point RP at the tip center of the tool die and defining the cutting speed *v_c_* and direction of motion of the tool, the tool has six degrees of freedom in 3D space, which move and rotate *V_x_* and *V_rx_* along the X-axis; move and rotate *V_y_* and *V_ry_* along the Y-axis; and move and rotate *V_z_* and *V_rz_* along the Z-axis. The constraints on its degrees of freedom are defined as *V_x_* = *v_c_*, *V_y_* = *V_z_* = *V_rx_* = *V_ry_* = *V_rz_* = 0, and the workpiece sides and the ground were completely fixed. The anisotropic nature of CF/PEEK (e.g., fiber breakage) requires that the fibers and the matrix be modeled separately. The carbon fibers were placed at a diameter of 7 µm and a pitch of 1 µm (in real-life materials, carbon fibers are randomly arranged; therefore, fixing their diameter and spacing is a modification introduced to simplify the modeling process and reduce the computational complexity, which differs from the actual arrangement). The matrix is set up with PEEK resin material parameters, and the tool is set up as a rigid body. Abaqus involves stable convergence when solving nonlinear problems, and it is necessary to ensure that each incremental step is smaller than the stability limit value. When damping is not taken into account, the conservative cell–cell estimation method is used. In order to speed up the computation time, the largest possible mesh size is selected. Considering that the diameter of the carbon fibers is 7 μm, and that the spacing between the fibers is 1 μm, the mesh size should be less than or equal to 1 μm; the limit of the stability time increment is in the order of 1 × 10^−9^; the mesh division size is 1 μm; and, after mesh checking, the minimum stability time increment is 1.21 × 10^−10^. The analysis step adopts the dynamics-display analysis, and the cell type of the PEEK resin matrix and the carbon fibers is set up as the eight-junction linear hexahedral cell C3D8R. The tool is set in contact with the material, the tangential and normal behaviors are considered to set up the tool and material, and the penalized contact method is set up.

Carbon fiber is modeled as an anisotropic linear elastic material due to its hardness and brittleness. The maximum stress failure criterion is implemented through the user-written VUMAT subroutine. When the stress exceeds the ultimate strength, the unit is removed. The maximum stress failure criterion is given in Equation (1) [31]:

(1) $$I_{F}=\max\left(\frac{\sigma_{C}}{C},\frac{\sigma_{T}}{T},\left|\frac{\gamma }{S}\right|\right)<1.0$$

*C*, *T*, and *S* denote the compressive, tensile, and shear failure stresses, respectively. Fiber fracture occurs when the value of *I_F_* exceeds Equation (1). The fiber damage factor, based on different loading conditions, is given by Equation (2) [31]:

(2) $$\begin{array}{l} \left\{\begin{array}{l} \mathrm{Longitudinal}\;\mathrm{tensile}\;\mathrm{load}\left(\sigma_{11}\geq 0\right)\;\;\;\;\;\;d_{t1}=\frac{\sigma_{11}}{X_{T}}\\ \mathrm{Transverse}\;\mathrm{tensile}\;\mathrm{load}\left(\sigma_{22}\geq 0\mathrm{or}\sigma_{33}\geq 0\right)\;\;d_{t2}=\frac{\sigma_{22}}{Y_{T}},d_{t3}=\frac{\sigma_{33}}{Y_{T}}\\ \mathrm{Longitudinal}\;\mathrm{compression}\;\mathrm{load}\left(\sigma_{11}<0\right)\;\;\;d_{c1}=\left|\frac{\sigma_{11}}{X_{C}}\right|\\ \mathrm{Transverse}\;\mathrm{compression}\;\mathrm{load}\left(\sigma_{22}<0\mathrm{or}\sigma_{33}<0\right)\;\;d_{c2}=\left|\frac{\sigma_{22}}{Y_{C}}\right|,d_{c3}=\left|\frac{\sigma_{33}}{Y_{C}}\right|\\ \mathrm{Internal}\;\mathrm{shear}\;\mathrm{load}\left(\gamma_{12}\neq 0\right)\;\;\;\;d_{s12}=\left|\frac{\gamma_{12}}{S}\right|\\ \mathrm{External}\;\mathrm{shear}\;\mathrm{load}\left(\gamma_{13}/\gamma_{23}\right)\;\;\;\;d_{s13}=\max\left(\left|\frac{\gamma_{13}}{S},\left|\frac{\gamma_{23}}{S}\right|\right|\right) \end{array}\right. \end{array}$$

*d* represents the damage factor in different directions, with the subscripts “*c*”, “*t*”, and “*s*” indicating compression, tension, and shear directions, marking damage initiation points. *σ* and *γ* represent the tensile (compression) and shear stresses. “S”, “X”, and “Y” denote the shear, longitudinal, and transverse strengths (X_T_: longitudinal tensile strength, X_C_: longitudinal compressive strength, Y_T_: transverse tensile strength, Y_C_: transverse compressive strength as shown in Appendix A).

PEEK is an isotropic elastoplastic material, with its elastic behavior defined by a linear elastic model and its plastic behavior described by the Johnson–Cook model once the stress exceeds the yield strength. The model incorporates the strain, strain rate, and temperature effects on the elasticity and plasticity, along with strain hardening, rate strengthening, and thermo-softening during plastic deformation. The Johnson–Cook model is expressed in Equation (3) [32,33]:

(3) $$\tilde{\sigma }=\left[A+B{\left({\bar{\varepsilon }}^{pl}\right)}^{n}\right]\left(1+Cnl\frac{{\overset{•}{\bar{\varepsilon }}}^{pl}}{{\overset{•}{\varepsilon }}_{0}}\right)\left[1-{\left(\tilde{T}\right)}^{m}\right]$$

where ${\bar{\varepsilon }}^{pl}$ is the effective plastic strain, ${\overset{•}{\bar{\varepsilon }}}^{pl}$ is the effective plastic strain rate, and *A*, *B*, *C*, *n*, and *m* are material parameters that are independent of each other (where *A* is the initial yield strength of the material, *B* is the strengthening coefficient, *C* is the strain rate sensitivity coefficient, *n* is the strain hardening index, and *m* is the temperature softening index). These parameters represent the yield stress, strain hardening, strain rate sensitivity, thermal softening, and hardening index at various strain rates and temperatures.

The Johnson–Cook damage model describes the onset of damage, as shown in Equations (4) and (5) [32,33].

(4) $${\bar{\varepsilon }}_{D}^{pl}=\left[d_{1}+d_{2}exp\left(-d_{3}\eta \right)\right]\left(1+d_{4}nl\frac{{\overset{•}{\bar{\varepsilon }}}^{pl}}{{\overset{•}{\varepsilon }}_{0}}\right)\left(1+d_{5}\tilde{T}\right)$$

(5) $$\tilde{T}=\frac{T-T\prime }{T_{m}-T\prime }\;\;\eta =\frac{P}{\bar{\sigma }}$$

Here, *d_1_*–*d_5_* represent the five failure parameters (*d*_1_ is the initial impairment variable, *d*_2_ is the damage amplitude variable, *d*_3_ is the damage strain impact factor, *d*_4_ is the strain rate sensitivity factor, and *d*_5_ is the temperature sensitivity factor), and ${\bar{\varepsilon }}_{D}^{pi}$ is the equivalent plastic strain at the initiation of damage. The material is considered undamaged until the equivalent plastic strain is greater than the damage initiation strain ${\bar{\varepsilon }}_{D}^{pi}$. *P* denotes the static pressure, and *η* is the stress triad dimension. This model is valid within the temperature range from room temperature (*T’*) to the melting point of the PEEK matrix (*T_m_*).

Second, let *ω* represent the cumulative damage function, as shown in Equation (6) [31]:

(6) $$\omega =\sum \frac{\Delta {\bar{\varepsilon }}^{pl}}{{\bar{\varepsilon }}_{0}^{pl}}$$

Here, the damage onset strain ${\bar{\varepsilon }}_{0}^{pl}$ is defined, and damage ${\bar{\varepsilon }}_{0}^{pl}$ is considered to begin only when this strain is reached. Specifically, damage initiates when *ω* = 1 (*D* = 0).

The fracture energy *G_f_* of the matrix during the damage evolution phase is defined using Hillerborg’s fracture energy criterion, as shown in Equation (7) [31]:

(7) $$G_{f}=\int_{{\bar{\varepsilon }}_{D}^{pl}}^{{\bar{\varepsilon }}_{f}^{pl}}L\sigma_{0}^{m}d{\bar{\varepsilon }}^{pl}$$

where ${\bar{\varepsilon }}_{D}^{pl}$ represents the equivalent plastic strain at the matrix failure, $\sigma_{0}^{m}$ is the yield stress, and *L* is the characteristic length.

The interface is the transition zone between carbon fibers and the matrix, including both chemical and mechanical bonding regions. The interface is crucial for adhesion and force transfer between the fibers and resin, affecting the mechanical properties of CF/PEEK composites. The cohesive model defines the contact properties between the carbon fiber and polyetheretherketone (PEEK), representing the interfacial relationship. The stiffness matrix is presented in Equation (8) [32]:

(8) $$t=\left\{\begin{array}{l} t_{n}\\ t_{s}\\ t_{t} \end{array}\right\}=\left(\begin{array}{ccc} k_{nn} & 0 & 0\\ 0 & k_{ss} & 0\\ 0 & 0 & k_{tt} \end{array}\right)\left\{\begin{array}{l} \delta_{n}\\ \delta_{s}\\ \delta_{t} \end{array}\right\}$$

*t_n_*, *t_s_*, *t_t_*, *δ_n_*, *δ_s_*, and *δ_t_* represent the stresses and traction displacements in the normal and shear directions. A quadratic stress criterion modeled the interface damage [34], with initiation occurring when the contact stress satisfied the conditions in Equation (9).

(9) $${\left\{\frac{t_{n}}{t_{n}^{0}}\right\}}^{2}+\left\{\frac{t_{s}}{t_{s}^{0}}\right\}+{\left\{\frac{t_{t}}{t_{t}^{0}}\right\}}^{2}=1$$

*t^0^* denotes the peak damage intensity, while $t_{n}^{0},t_{s}^{0},t_{t}^{0}$ represent the stress intensities in the normal and shear directions, respectively, as the maximum values in each direction. The energy-based Benzeggagh–Kenane (B-K) criterion models damage evolution after initiation. The total fracture energy is expressed by Equation (10) [35]:

(10) $$G^{C}=G_{n}^{C}+\left(G_{s}^{C}-G_{n}^{C}\right){\left(\frac{G_{s}+G_{t}}{G_{n}+G_{s}+G_{t}}\right)}^{\eta }$$

$G_{n}^{C}$ and $G_{s}^{C}$ represent the fracture energies in the normal and shear directions, respectively; the work performed by the traction stress in these directions is denoted by $G_{n},G_{s}$ and $G_{t}$, respectively; and *G_t_* is the B-K mixed-mode interaction index. The property parameters for carbon fiber, polyetheretherketone, and the interface in CF/PEEK composites are shown in Table 2.

## 3. Materials and Methods

### 3.1. Test Materials

The CF/PEEK composite specimens consisted of 38 layers, each 0.135 mm thick, with a fiber diameter of 7 microns and a mass fraction of about 50%. The layup angles were 0°, 45°, 90°, and 135°. The unidirectional plate was fabricated using a hot molding process, then cut into 60 mm × 30 mm × 5 mm pieces by a water jet. The specimens were polished using graded sandpaper (P800, P1200, P2000, Shuriken Sandpaper, Hainan, China.) to achieve a surface roughness (Ra) < 6.4 μm, as measured after polishing [36]. Optical microscopy (Keens, Osaka, Japan) confirmed the absence of visible damage, including fiber-matrix debonding and microcracks. Table 3 presents the detailed properties of the CF/PEEK specimens.

### 3.2. Test System

**Figure 2:**
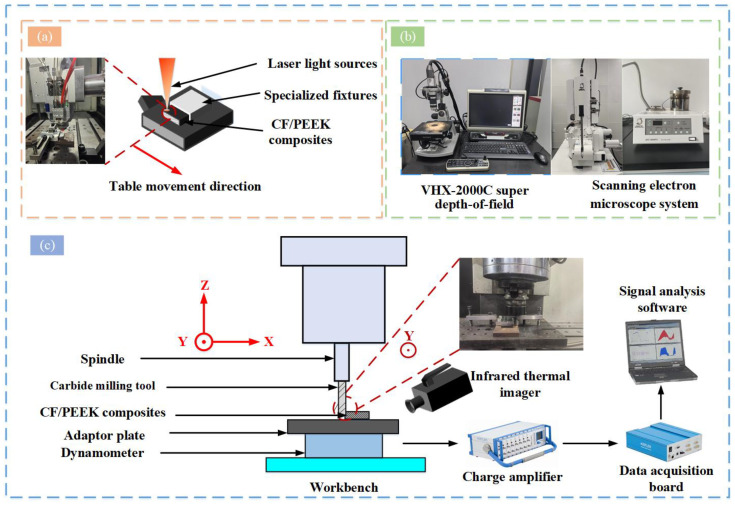
Schematic diagram of test and inspection equipment: (**a**) Schematic diagram of laser scanning, (**b**) main inspection equipment, and (**c**) test device.

Laser composite milling experiments were conducted to examine the influence of the spindle speed and fiber milling angle on CF/PEEK processing and its mechanisms. The CF/PEEK unidirectional plate was first scanned with a continuous laser, followed by CNC milling to remove the heat-affected zone, improving the surface quality and reducing tool wear. The experimental setup for laser scanning, shown in Figure 2a, consisted of a six-axis robotic arm equipped with an IPG continuous laser head (3000 W, 1064 nm) and a CNC vertical machining center. The CF/PEEK unidirectional plate was positioned horizontally, with the laser applied vertically to the upper edge, ensuring side visibility of the heat-affected zone. The laser head-to-surface distance was adjusted using the 6-axis robotic arm to control the angle of incidence and spot radius. The laser spot radius must be smaller than the unidirectional plate thickness, with the scanning speed controlled by the vertical machining center. An air-cooling system was used during laser irradiation. Afterward, the heat-affected zone depth was measured with a VHX-2000C super depth-of-field microscope (Figure 2b), ensuring that it was less than the workpiece thickness. Following previous research [37], the laser power was set to 400 W and the feed rate to 250 mm/min. Detailed parameters are provided in Table 4.

An experimental study was conducted using a four-flute carbide cutter (diameter: 8 mm, front angle: 7°, back angle: 9°, helix angle: 45°) in the milling process. The tool was attached to the spindle of a vertical machining center. The workpiece was fixed with a custom fixture in a cutting force measurement system, which included a Swiss Kistler force sensor, the Dynoware (DynoWare3.2.2.0-1.0) software, and an FLIR T630sc camera (FLIR Systems Inc., Wilsonville, OR, USA) for real-time temperature measurements. The system was placed on a vertical machining center for cutting force measurements, as shown in Figure 2c. A downward milling process minimized burr formation and fiber delamination, with a horizontal cut width of 5.0 mm and a radial depth of 3.0 mm (parameters in Table 4). The machined surfaces and subsurface damage were observed using a VHX-2000C microscope (Keens, Osaka, Japan) and a JSM-IT800 SEM (Nippon Electronics Corporation, Tokyo, Japan). The samples were gold-sputtered before the SEM inspection. Each experiment was repeated three times to minimize random error.

## 4. Results and Discussion

The laser-assisted milling model data were compared with the experimental results. The milling parameters are defined as follows: CM/LAC for conventional milling/laser-assisted milling, 0/45/90/135 for cutting angles of 0°, 45°, 90°, and 135°, and 500/2000/4000 for spindle speed (r/min) (see Appendix A.1 for details).

### 4.1. Cutting Force Analysis

The cutting forces in the X, Y, and Z directions were recorded. *Fx* and *Fy* denote the feed and axial cutting forces in the machining plane, while Fz is the perpendicular component. The total cutting force, *F*, was then simplified as follows. Since the machined surface quality and fiber damage were primarily influenced by *Fx*, both the total cutting force, *F*, and *Fx* were analyzed in detail.

(11) $$F=\sqrt{F_{x}^{2}+F_{y}^{2}+F_{z}^{2}}$$

**Figure 3:**
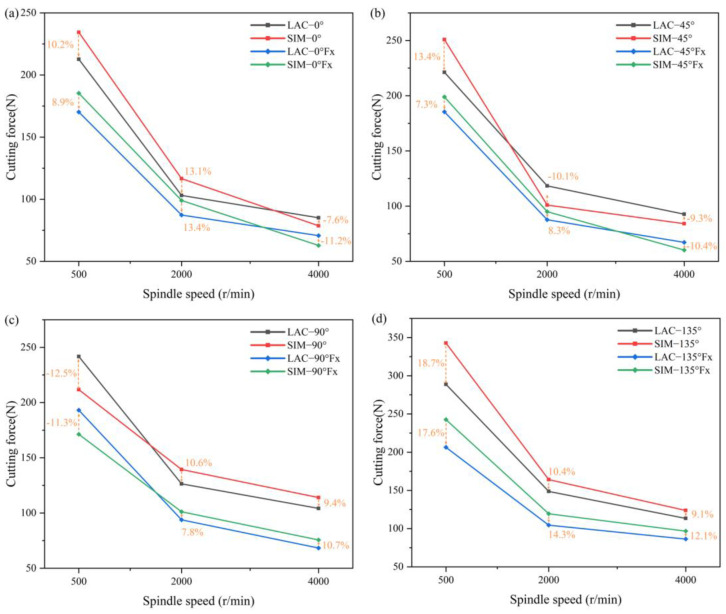
Comparison of laser-assisted milling cutting force simulation experiments: (**a**) 0° milling angle, (**b**) 45° milling angle, (**c**) 90° milling angle, and (**d**) 135° milling angle.

**Figure 4:**
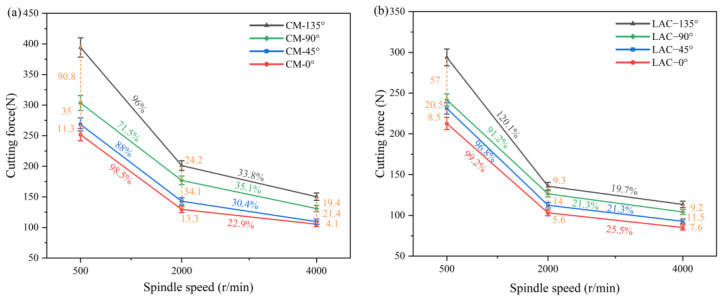
Experimental comparison of cutting forces in milling: (**a**) conventional machining, (**b**) laser-assisted machining.

Figure 3 compares the cutting forces at four milling angles for the simulated and experimental results of laser-assisted milling. The analysis shows that, during laser-assisted machining, the cutting force at 135° was significantly higher than that at 90°. In addition, the cutting forces at the cutting angles of 90°, 45°, and 0° showed very little variation, and the simulation and experimental trends were consistent. The maximum simulation–experiment error in the total cutting force was 18.7%, which was less than 20% for the laser-assisted condition at a cutting angle of 135° and 500 r/min. Figure 4 shows the experimental data of the cutting force under various machining conditions. The results show that the cutting force error for conventional machining was large, while the stability of laser-assisted milling was better. Laser-assisted milling resulted in smoother machine loading and a significant reduction in vibration. This phenomenon correlates with the reduction in cutting force, further validating the advantages of laser-assisted milling in improving machining stability.

Figure 4 shows a clear trend in the combined cutting forces for conventional and laser-assisted milling. The cutting forces at a cutting angle of 135° were significantly higher than those at a cutting angle of 90° and exceeded those at a cutting angle of 45°. The difference in the cutting force at 45° and 0° was small. The integrated cutting force reached its maximum value when the spindle speed was 500 r/min, and then decreased gradually with an increase in speed. Increasing the milling angle increased the contact area between the cutting edge and the workpiece and reduced the cutting angle inside the material. This geometric change enhanced the cutting action of the tool, resulting in higher material removal rates and greater cutting forces. As the spindle speed increased, the strain rate of the PEEK resin improved its mechanical properties, which enhanced the strength of the fibers, making them less prone to bending and more prone to breaking, ultimately reducing the cutting force. In addition, the significant increase in heat generated during the cutting process raised the local temperature of the material, which reduced the hardness and cutting resistance, further reducing the cutting force.

**Figure 5:**
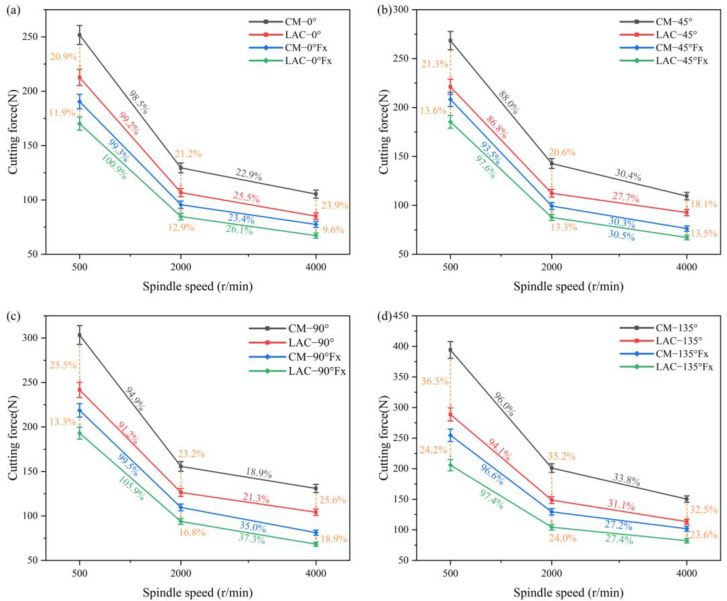
Cutting forces in conventional and laser-assisted milling: (**a**) 0° cutting angle, (**b**) 45° cutting angle, (**c**) 90° cutting angle, and (**d**) 135°cutting angle.

As shown in Figure 5, laser composite milling significantly reduced the cutting forces at the 90° and 135° cutting angles, while the cutting forces at the 0° and 45° cutting angles were not significantly reduced. In Figure 5c, laser composite milling reduced the total cutting forces by an average of 24.8% and the feed-side cutting forces by 16.3% at a 90° cutting angle compared to conventional milling. At a 90° cutting angle, laser composite milling ablated most of the PEEK resin, reducing the material removal rate and cutting forces (ablation of PEEH resin is the removal of material by thermal decomposition of the surface by laser heating). The variation in the feed-cutting force at the 90° cutting angle was greater than in conventional milling. The ablation of PEEK resin weakens the strain rate, enhancing the mechanical behavior and increasing the cutting force. As shown in Figure 5d, laser composite milling reduces the cutting forces at a cutting angle of 135° compared to conventional milling. Specifically, the total cutting force was reduced by 34.7%, and the feed-cutting force was reduced by 23.9%. Conventional milling at a cutting angle of 135° requires a higher bending fracture energy than the other angles, resulting in higher cutting forces. During the laser ablation process, the heat-affected zone mainly extends along the axial direction of the fiber due to the significantly higher thermal conductivity of the carbon fiber in the axial direction (50 W/mK) than in the radial direction (5 W/mK) [38]. The direction of the laser scanning speed is at an acute angle to the fiber axial heat conduction at the cutting angle of 135°, which promotes the expansion of the laser energy, resulting in an order of the heat-affected zone of 135° > 90° > 45° > 0° under the same laser parameters. As a result, in laser composite milling, the removal of CF/PEEK is minimized at a cutting angle of 135°, resulting in the fibers being susceptible to shear fracture at this angle, which significantly reduces the cutting force [39,40]. This process optimizes the fiber fracture behavior and slows down the increase in cutting forces due to increased material removal. The increase in the feed-direction cutting force is more gradual than in conventional milling.

### 4.2. Cutting Temperature Analysis

**Figure 6:**
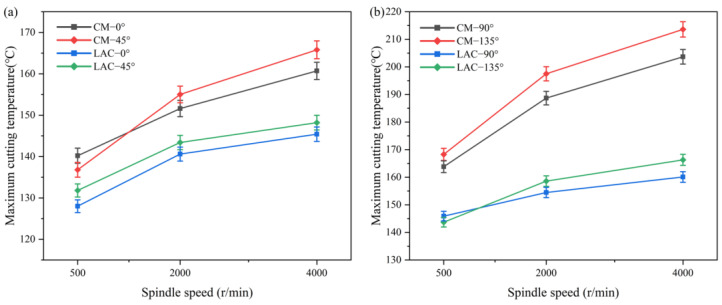
Cutting temperature data under different machining processes: (**a**) 0° vs. 45°; (**b**) 90° vs. 135°.

Figure 6 presents the maximum cutting temperature for various cutting parameters. Laser composite milling lowers the cutting temperatures relative to conventional methods. In conventional milling, cutting heat arises primarily from tool–workpiece friction [41]. Figure 6a,b show that, at the cutting angles of 0° and 45°, laser composite milling did not notably reduce the cutting temperature compared to conventional milling. However, at the cutting angles of 90° and 135°, laser composite milling effectively reduces the cutting temperature. At a cutting angle of 135°, the maximum cutting temperature reached 213.6 °C, higher than the 203.7 °C at a cutting angle of 90°. The temperature also increased with the spindle speed, in line with other studies [42]. Two key factors influence temperature variations during milling: first, the feed-cutting force peaks at a cutting angle of 135°. Although this cutting force decreases with the spindle speed, the heat generated during the process remains higher than the reduction in work loss. Second, fiber bending is more pronounced at 135° than at a cutting angle of 90°, causing a stronger rebound effect on the material. The rebound effect intensifies with a higher spindle speed, enlarging the contact area between the tool and the workpiece. This change increases the friction, enhancing heat buildup and significantly raising the temperature during cutting. Under laser composite milling, the maximum cutting temperature remained around 163 °C for both the cutting angles of 135° and 90°. As the milling speed increases, the variation in the maximum cutting temperature becomes minimal and levels off. Laser ablation removes most resin, reducing the cutting force and frictional heat. The heat from fiber breakage and chip deformation increases. The heat does not increase significantly with the spindle speed. Instead, an increased spindle speed increases the proportion of fibers undergoing shear fracture, forming longer fibers. During separation, these longer fibers carry away heat, reducing the cutting temperature.

### 4.3. Machined Surface Damage

**Figure 7:**
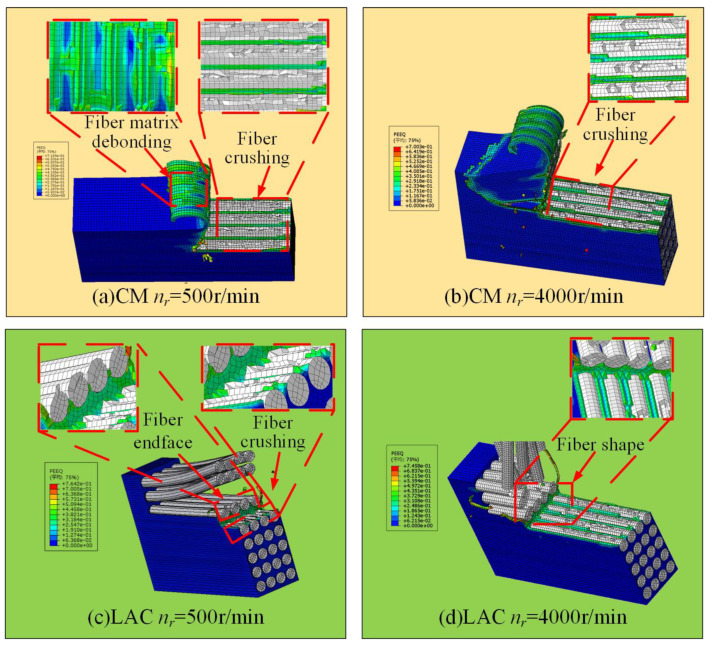
Milling with 0° cutting angle machining surface simulation results: (**a**) CM *n_r_* = 500 r/min; (**b**) CM *n_r_* = 4000 r/min; (**c**) LAC *n_r_* = 500 r/min; (**d**) LAC *n_r_* = 4000 r/min.

**Figure 8:**
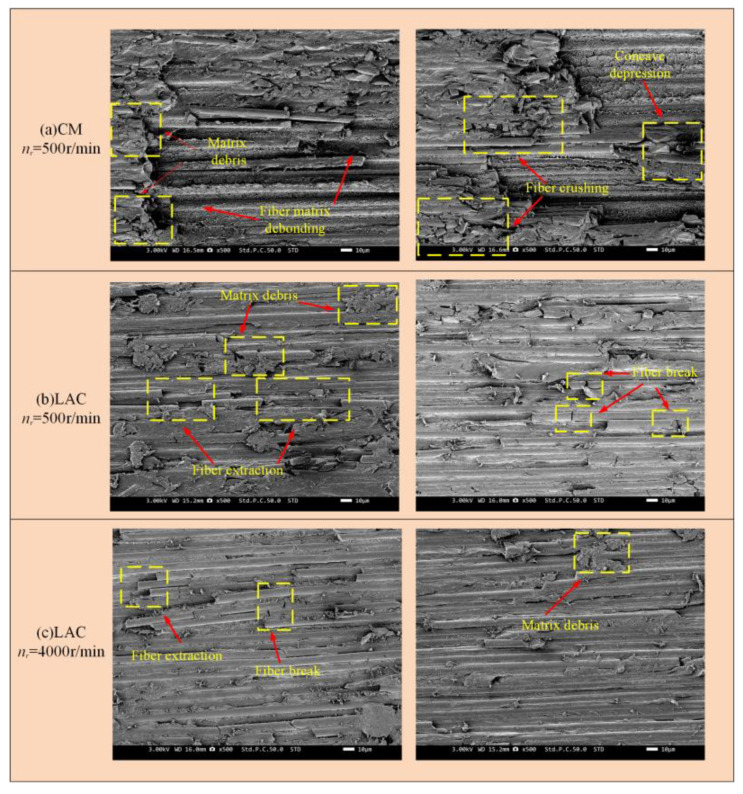
Milling with 0° cutting angle machining surface: (**a**) CM *n_r_* = 500 r/min; (**b**) LAC *n_r_* = 500 r/min; (**c**) LAC *n_r_* = 4000 r/min.

Scanning electron microscopy was used to observe the milled CF/PEEK surface, revealing the surface damage, fiber morphology, and fracture mechanisms. Figure 7 illustrates the surface damage characteristics simulated at a 0° cutting angle. As the tool contacted the workpiece, cracks formed on the carbon fiber surface, leading to brittle fracture and material removal. As the carbon fiber was removed, debonding between the fiber and the substrate occurred. As the milling tool progressed, in addition to fiber/substrate debonding, some fibers were pulled out, creating bare fiber grooves. This occurs because, at a 0° cutting angle, the tool’s front face exerts axial extrusion on the fiber, causing the upper layer of fibers to bend upward. When the bending reaches the fiber’s limit, debonding between the fiber and substrate occurs. As the rotational speed increases, the surface damage decreases. In contrast, during laser-assisted milling, the fiber fracture surface was smoother, with only partial fiber rupture, which was consistent with the experimental results (Figure 8). Figure 8 shows broken fibers and resin matrix debris on the surface at a 500 r/min spindle speed, with the fractures exhibiting an extruded morphology. The presence of pits on the surface indicated significant fiber extrusion by the rear cutter surface, causing fiber crushing, fracture, and debonding at the fiber–matrix interface. As the spindle speed increased, the surface quality gradually improved. In laser-assisted milling, the surface finish improved significantly compared to conventional milling, with minimal fiber fracture, resin fragmentation, and fiber pull-out, and these undesirable phenomena occurred less frequently. At a 500 r/min spindle speed, the surface damage mainly manifested as fiber crushing, bending, and segmental fracture. When the fiber reached its tensile strength limit, short fiber segments continued to transfer stress through the matrix, leading to stress concentration at the fiber–matrix interface [43]. This stress concentration caused the further breakage of short fibers. Additionally, friction from the tool’s rear blade surface may cause short fiber pull-out and fracture, leading to holes in the machined surface. Fiber crushing, bending, and fracture decreased as the spindle speed increased.

**Figure 9:**
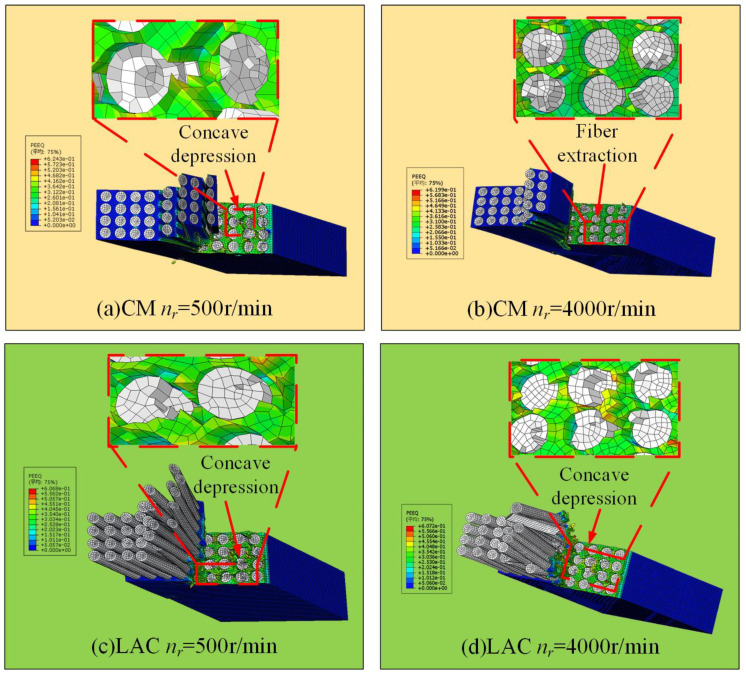
Milling with 45° cutting angle machining surface simulation results: (**a**) CM *n_r_* = 500 r/min; (**b**) CM *n_r_* = 4000 r/min; (**c**) LAC *n_r_* = 500 r/min; (**d**) LAC *n_r_* = 4000 r/min.

**Figure 10:**
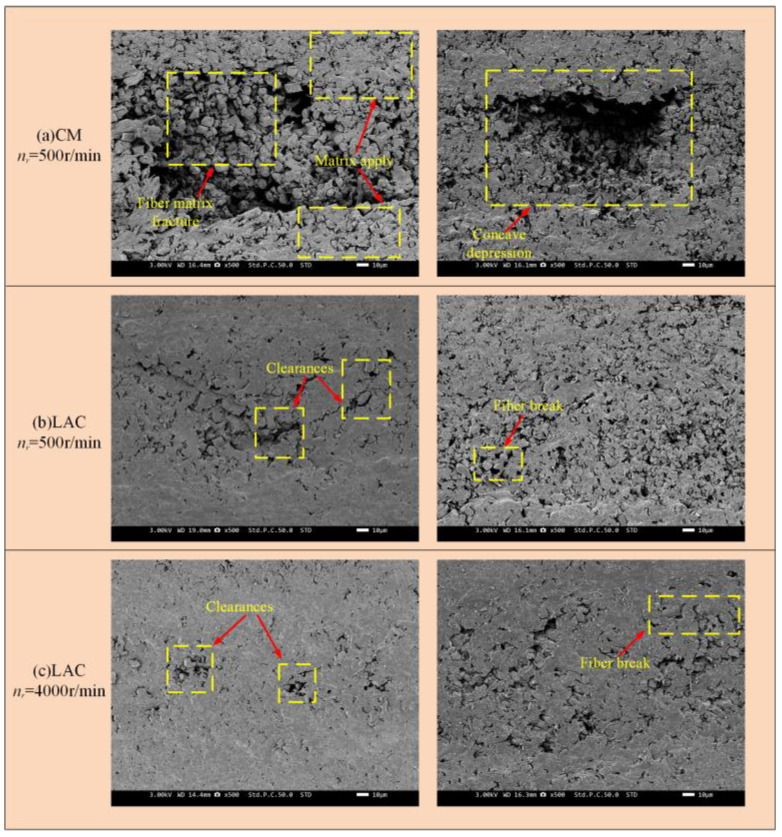
Milling with 45° cutting angle machining surface: (**a**) CM *n_r_* = 500 r/min; (**b**) LAC *n_r_* = 500 r/min; (**c**) LAC *n_r_* = 4000 r/min.

Figure 9 presents the simulation results of the surface damage on CF/PEEK composites machined at a 45° cutting angle. The model analysis revealed significant cratering between the matrix and fibers during conventional milling, which decreased as the spindle speed increased. No significant cratering was observed after laser-assisted milling. Figure 10 presents SEM images of the surface damage on CF/PEEK composites machined at a 45° cutting angle. At a 500 r/min spindle speed, significant smearing appeared on the matrix and fiber surfaces, accompanied by surface craters from missing matrix and fibers, as well as numerous craters between them. This occurred because of the 45° cutting angle; the fibers underwent significant shear fracture and compressive failure. Due to the cutting angle of 45°, the fibers had a diagonal squeezing effect on the front face of the tool, and the fibers bent backward and experienced compression damage at the contact area. Exceeding the fiber’s yield limit induced an inclined crack in the subsurface, perpendicular to its axis. Under bending stress, this inclined crack expanded into a shear surface, leading to fiber shear fracture. Shear fracture also occurred in the subsurface region of the fiber. If the shear surface is shallow, the tool may pull out most fractured fibers, forming surface craters. As the spindle speed increased, the surface quality of the composite improved. After laser-assisted milling, the CF/PEEK composites showed no significant pits, only a few voids and irregular fractures in individual fibers. No significant changes were observed in the machined surfaces at varying spindle speeds.

**Figure 11:**
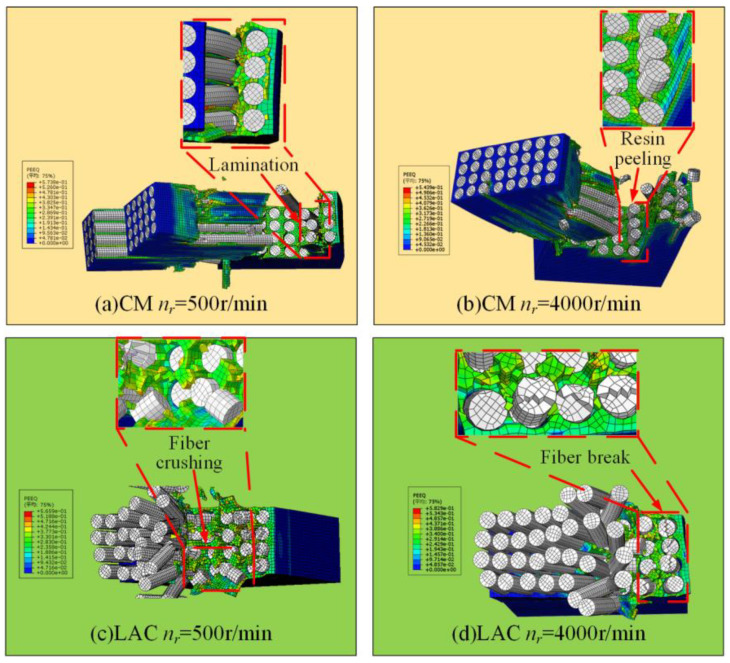
Milling with 90° cutting angle machining surface simulation results: (**a**) CM *n_r_* = 500 r/min; (b) CM *n_r_* = 4000 r/min; (**c**) LAC *n_r_* = 500 r/min; (**d**) LAC *n_r_* = 4000 r/min.

**Figure 12:**
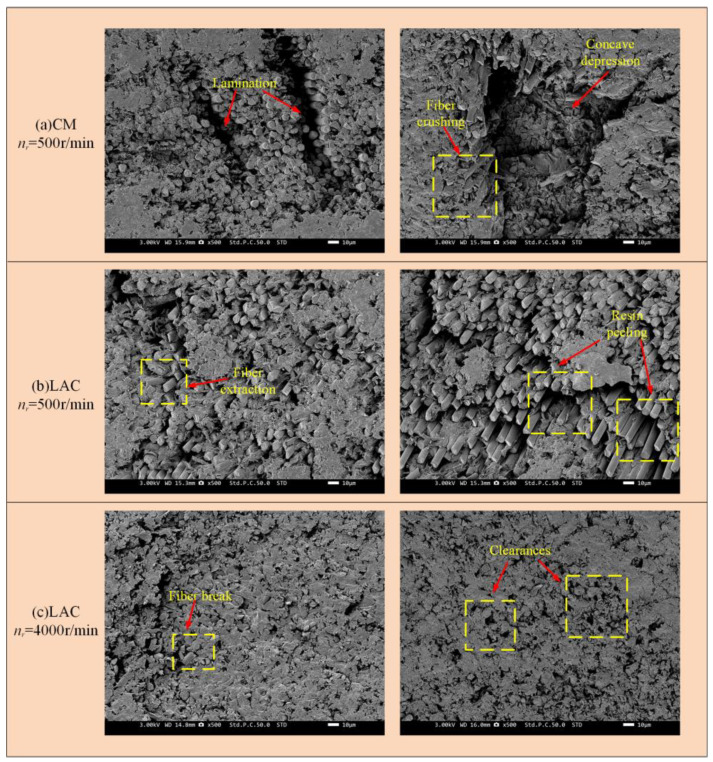
Milling with 90° cutting angle machining surface: (**a**) CM *n_r_* = 500 r/min; (**b**) LAC *n_r_* = 500 r/min; (**c**) LAC *n_r_* = 4000 r/min.

Figure 11 shows the surface damage patterns from simulations at a 90° cutting angle. Under conventional machining conditions, fibers were significantly crushed and broken, with more severe bending. Laser-assisted cutting produced a flatter fiber fracture surface, showing a short fiber morphology and partial fiber pull-out, as confirmed by the experimental results (Figure 12). In Figure 12, at a spindle speed of 500 r/min, the machined surface showed delamination, with significant broken fibers and resin residues. The fiber arrangement was relatively tight, and the fracture exhibited extrusion crushing, indicating more severe extrusion and crushing of the fibers. During laser composite milling, the machined surface was flatter than that of conventional machining, with no cracks. However, slight fiber pull-outs and resin shedding were observed. At a spindle speed of 500 r/min, residual fibers exhibited a short strip structure. As the spindle speed increased, the fiber arrangement loosened and the fractures flattened. A simulation analysis of the fracture mode of fibers after laser composite milling is shown in Figure 12 at a 90° cutting angle. First, the fiber underwent compressive stress at the cutter tip, leading to fracture. At the fiber fracture point, the upper fiber underwent both upward and downward tension, while the front was subjected to forward extrusion and lateral friction from the tool face. As a result, the fibers in this region were gradually crushed, exhibiting localized breakage, typical of compression fracture failure. The lower fiber underwent compressive stress from the cutter tip perpendicular to the fiber axis, causing bending and crack extension and leading to under-face damage as a bending fracture.

**Figure 13:**
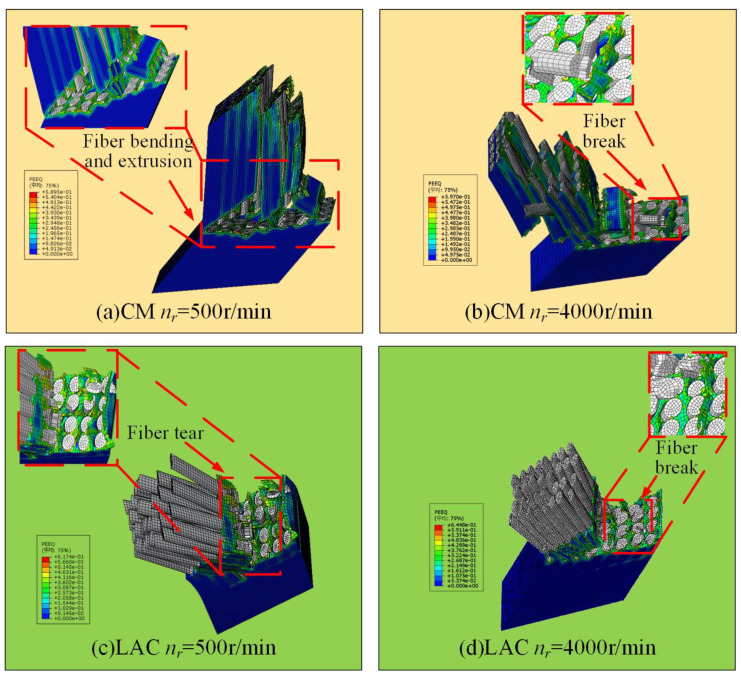
Milling with 135° cutting angle machining surface simulation results: (**a**) CM *n_r_* = 500 r/min; (**b**) CM *n_r_* = 4000 r/min; (**c**) LAC *n_r_* = 500 r/min; (**d**) LAC *n_r_* = 4000 r/min.

**Figure 14:**
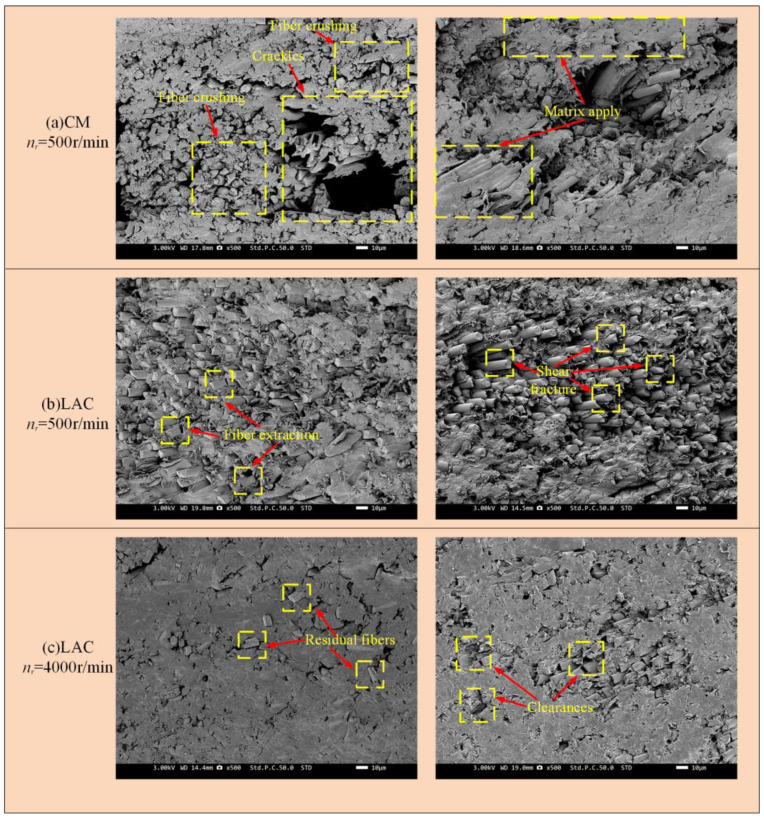
Milling with 135° cutting angle machining surface: (**a**) CM *n_r_* = 500 r/min; (**b**) LAC *n_r_* = 500 r/min; (**c**) LAC *n_r_* = 4000 r/min.

Figure 13 presents the surface damage morphology from the simulation at a 135° cutting angle. Under conventional conditions, the machined surface mainly exhibited an extruded and broken morphology, with numerous cracks formed due to fiber bending and matrix shift. In laser-assisted milling, the fiber fractures in the first half exhibited a tearing pattern, while those in the second half were flatter. Figure 14 presents the SEM image at a 135° cutting angle. Under conventional cutting conditions, the surface cracks were more severe, with the fiber fractures exhibiting an extruded and crushed morphology, and the fibers were tightly packed. At a spindle speed of 500 r/min, surface cracking was particularly severe, with obvious matrix smearing and more prominent fiber breakage. However, as the spindle speed increased, the surface damage decreased. In laser composite milling, no significant cracking occurred, with only minimal fiber pull-out. The fiber fracture exhibited a shear pattern, with residual fibers forming short strips. Moreover, no significant differences were observed between the machined surfaces at varying spindle speeds. At the 135° cutting angle, fiber fractures exhibited a bending-dominant mode [44], requiring more energy and cutting force and resulting in greater surface damage. At a cutting angle of 135, the fibers underwent compression fracture and in-surface shear damage. When the tool contacts the fibers, the force exerted by the tip resembles three-point bending, causing fibers on both sides to converge towards the tip. Fiber bending is more pronounced at the upper tool tip due to shorter fibers and the lack of binding in the lower fibers. As the upper fibers bend, noticeable compression–shear fracture occurs at the tool tip. The upper fibers are sheared by extrusion from the tool face, causing in-plane shear between the fibers and substrate and leading to fiber extrusion along the tool. As the cutting depth increases, the bending of the lower fibers leads to a higher compressive stress at the tool tip and shear stress at the rear tool face, causing crack expansion, debonding between the fibers and the substrate, and the formation of a void on the material surface.

## 5. Conclusions

This study examined the cutting force, temperature, surface damage, and material removal in CF/PEEK composites during laser-assisted milling at different spindle speeds and fiber orientations. The fiber fracture process was simulated and compared with experimental results. The key findings include the following:

(1) Laser-assisted milling significantly reduced both the total and feed-cutting forces in CF/PEEK composite machining compared to conventional milling. The greatest reduction in cutting forces occurred at a fiber orientation angle of 135°, followed by 90°, 45°, and 0°. Laser treatment enhanced the fiber fracture pattern and reduced the cutting forces.
(2) At a constant feed rate, the milling force decreased sharply (by over 90%) with increasing spindle speed. Above 2000 r/min, the milling force reduction stabilized. Increasing the spindle speed at a constant feed rate reduced the feed per tooth, greatly impacting the milling force. Consequently, beyond 2000 r/min, minor variations in the feed per tooth led to a leveling off of the cutting force reduction.
(3) Laser-assisted milling lowered the cutting temperatures relative to conventional milling. In contrast to conventional milling, laser-assisted milling exhibited the greatest temperature reduction at the 90° and 135° fiber orientations, with this effect intensifying as the spindle speed increased. In laser-assisted milling, lower cutting forces and frictional heat between the tool and workpiece amplified the heat from fiber breakage and chip deformation. Additionally, some heat was carried away as the fiber chips separated, further lowering the cutting temperature.
(4) A simulation model for laser-assisted milling of microcrystalline cuts with varying fiber orientations was developed, and the fracture modes were analyzed. An experimental analysis of laser-assisted milling with varying fiber orientations confirmed that the micro-deformation removal mechanism aligned with the simulation results. At a 0° cutting angle, fractures occurred primarily through bending; at 45°, it involved bending-shear fractures; at 90°, it was dominated by compression fractures; and at 135°, it followed a compression-shear fracture mode. Thus, the proposed microscopic cutting simulation model effectively simulated the material’s microscopic fiber-removal mechanism. (While the model was able to make accurate predictions for known data ranges, the results may lose accuracy when extrapolated for data beyond this range. Therefore, it is recommended that the extrapolated results of the model be used with caution in real-world applications and that further validation or extension of the data be considered before applying it to unknown data ranges.)
(5) This study provides theoretical support and technical guidance for the practical processing of laser-assisted CF/PEEK composites. In future research, this study will serve as the foundation for the further exploration of fiber changes and optimization strategies for process parameters, ultimately guiding the high-quality, efficient processing of CF/PEEK composites.

## Figures and Tables

**Table 1:** Simulation parameters.

Parameters	Values	Parameters	Values
*l* × *w* × *h*	77 × 32 × 100 μm	*γ*	15°
*φ*	5°	*r_e_*	1.5 μm
*h_t_*	40 μm	*a_c_*	30 μm
*l_1_*	25 μm	*v_c_*	12.56, 50.27, 100.53 m/min

**Table 2:** Properties of CF/PEEK composite materials [32].

Material	Property	Values
Carbon fiber	Elastic constants	*E*_11_ = 235 GPa, *E*_22_ = 14 GPa
		*E*_33_ = 14 GPa
		*v*_12_ = *v*_13_ = *v*_23_ = 0.25
		*G*_12_ = 28 GPa, *G*_13_ = 28 GPa
		*G*_23_ = 5.5 GPa
	Longitudinal strength	*X*_t_ = 3590 MPa, *X*_c_ = 2700 MPa
	Transverse strength	*Y*_t_ = 3590 MPa, *Y*_c_ = 2700 MPa
PEEK	Elastic constants	*E*_33_ = 4.1 GPa, *v* = 0.38
	J-C plastic parameter A, B, C, *n*, m	*A* = 132 MPa, *B* = 10 MPa, *C* = 0.035
		*m =* 0.7, *n* = 1.2
	J-C failure parameter d1~d5	0.05, 1.2, 0.254, −0.009,1
Interface	Cohesive stiffness	*K* = 6.4 × 10^5^ MPa/mm
	Normal strength	$t_{n}^{0}$ = 43 MPa
	Shear strength	$t_{s}^{0}$ = $t_{t}^{0}$ = 50 MPa

**Table 3:** Material properties [3,21].

Material	Parameters	Values
CF/PEEK	Density	1.550 g/cm^3^
	Tensile strength	2200 MPa
	Compressive strength	1200 MPa
	Bending strength	2000 MPa
	Bending modulus	130 GPa
	Interlaminar shear strength	90 MPa
	Transverse Poisson’s ratio	0.25
	Longitudinal Poisson’s ratio	0.38
Carbon fiber (50%)	Density	1.80 g/cm^3^
	Tensile strength	4900 MPa
	Tensile modulus	230 GPa
Polyetheretherketone resins (50%)	Density	1.3 g/cm^3^
	Tensile strength	95 MPa
	Melting temperature	343 °C
	Glass transition temperature	143 °C
	Elongation at break	15%

**Table 4:** Experimental parameters in co-processing.

Parameters	Values	Parameters	Values
Laser power	400 W	Shank diameter	8 mm
CNC movement speed	250 mm/min	Shank length	60 mm
CNC spindle speed	500, 2000, 4000 r/min	Blade length	20 mm
CNC feed speed	250 mm/min	Tool rake angle	15°
Fiber cutting angle	0°, 45°, 90°, 135°	Tool trailing angle	5°
Horizontal cut width	5 mm	Tool helix angle	45°
Radial depth of cutting	3 mm	Knife blade	4

## Data Availability

All the data and models generated or used during this study appear in the submitted article.

## Appendix A

### Appendix A.1

The subscripts in Equation (2) are interpreted as follows: (1) denotes the longitudinal direction, i.e., the direction of the carbon fibers; (2) denotes the transverse direction, perpendicular to the direction of the carbon fibers, in the in-face direction of the composite material; and (3) denotes the thickness direction (the outer direction), perpendicular to the direction of the composite material layup, i.e., the out-of-face direction. Therefore, the longitudinal load is *σ*_11_, the transverse load is *σ*_22_ and *σ*_33_, the internal shear load is *γ*_12_, and the external shear load is *γ*_13_ and *γ*_23_.

### Appendix A.2

The in-plane modulus of elasticity (*E*_11_) represents the stiffness along the fiber direction and is typically higher in the in-plane direction due to the high rigidity of carbon fibers. In this study, the in-plane modulus of elasticity was 235 GPa, which reflects the rigidity of the fibers and directly influences material deformation under cutting forces.

The out-of-plane modulus of elasticity (*E*_22_, *E*_33_) refers to the stiffness perpendicular to the fiber direction. The out-of-plane modulus is typically lower due to the matrix material (PEEK). In this study, the out-of-plane modulus was 14 GPa, indicating that the material was relatively weak in resisting deformation perpendicular to the fiber direction.

The in-plane Poisson’s ratio (*v*_12_) and the out-of-plane Poisson’s ratios (*v*_13_, *v*_23_, *v*) describe the material’s deformation properties along and perpendicular to the fiber direction, respectively. A smaller in-plane Poisson’s ratio indicates greater rigidity in the fiber direction, while a larger out-of-plane Poisson’s ratio indicates a higher deformability of the matrix material. In this study, an in-plane Poisson’s ratio (*v*_12_) of 0.25 and an out-of-plane Poisson’s ratio (*v*) of 0.38 were used.

These parameters significantly influence the material’s stress distribution and deformation behavior during the cutting process. Specifically, the in-plane and out-of-plane elastic moduli, as well as Poisson’s ratio, directly impact the cutting performance and machinability.

### Appendix A.3

Since the laser spot radius was 3 mm and the laser power was 400 W, the heat flux was about 5.66 × 10^7^ W/m^2^.

The temperature rise under short-time laser action was estimated using the following simplified equation [45]:

$$T_{s}=T_{\infty }+n\frac{q}{\rho c_{p}\sqrt{\pi \alpha }}$$

where *T_s_* is the surface temperature, *T_∞_* is the room temperature, *q* is the laser heat flux (W/m^2^), *n* is the absorption rate of the laser by the material (0.3), *ρ* is the density of the material (kg/m^3^), *c_p_* is the specific heat capacity of the material (1300 J/kg·K), α is the thermal diffusivity of the material (m^2^/s), and *k* is the thermal conductivity (50 W/mK).

Substituting the parameters into the formula yields a *T_s_* of about 950 °C > *T_m_*.

### Appendix A.4

**Table A1:** Experimental data.

Cutting Angle	Spindle Speed (r/min)	Total Cutting Force F (N)			Feed-Cutting Force F × (N)			Cutting Temperature (°C)	
		CM	LAC	Emulate	CM	LAC	Emulate	CM	LAC
0°	500	251.7	212.7	234.4	190.5	170.2	185.3	140.2	128.0
	2000	129.5	103.1	116.6	95.6	87.3	99.0	151.6	140.6
	3000	105.4	85.1	78.6	77.5	70.7	62.8	160.7	145.4
45°	500	268.4	221.2	250.8	208.3	185.4	198.9	136.8	131.8
	2000	142.8	112.4	101.2	99.4	87.7	95	155.1	143.4
	4000	109.5	92.7	84.1	76.3	67.2	60.2	165.8	148.2
90°	500	303.4	241.7	211.7	218.7	193.1	171.3	163.8	145.9
	2000	176.9	126.4	139.4	109.6	93.8	101.1	188.7	154.5
	4000	130.9	104.2	114	81.2	68.3	75.6	203.7	160.1
135°	500	394.2	293.7	342.7	254.6	206.3	242.6	168.3	143.7
	2000	201.1	140.7	164.2	129.5	104.5	119.4	197.5	158.6
	4000	150.3	113.4	123.7	101.8	86.3	96.7	213.6	166.3

## References

[1] Hassan E.A., Ge D., Zhu S., Yang L., Zhou J., Yu M. (2019). Enhancing CF/PEEK composites by CF decoration with polyimide and loosely-packed CNT arrays. Compos. Part A Appl. Sci. Manuf..

[2] Hassan E.A., Yang L., Elagib T.H., Ge D., Lv X., Zhou J., Yu M., Zhu S. (2019). Synergistic effect of hydrogen bonding and π-π stacking in interface of CF/PEEK composites. Compos. Part B Eng..

[3] Cao H., Song Y., Wu B., Wang K., Qu D. (2021). A force model of high-speed dry milling CF/PEEK considering fiber distribution characteristics. J. Manuf. Process..

[4] Lekrine A., Belaadi A., Dembri I., Jawaid M., Ismail A.S., Abdullah M.M., Chai B.X., Al-Khawlani A., Ghernaout D. (2024). Thermomechanical and structural analysis of green hybrid composites based on polylactic acid/biochar/treated *W. filifera* palm fibers. J. Mater. Res. Technol..

[5] Babu J., Basavarajappa S., Blass D., Blümel S., Chatelain J.F., Cong W., Díaz-Álvarez J., Dilger K., Feito N., Fischer F. (2015). Machinability of Fibre-Reinforced Plastics.

[6] Cao S., Zhang K., Hou G., Luo B., Cheng H., Li Y., Li X., Liu C. (2022). Experimental analysis of entrance and exit damage mechanism affected by the structural dynamic deformation characteristics during drilling of thin-walled CFRP. Thin-Walled Struct..

[7] Cao H., Liu L., Wu B., Gao Y., Qu D. (2021). Process optimization of high-speed dry milling UD-CF/PEEK laminates using GA-BP neural network. Compos. Part B Eng..

[8] Su F., Yuan J., Sun F., Wang Z., Deng Z. (2018). Analytical cutting model for a single fiber to investigate the occurrences of the surface damages in milling of CFRP. Int. J. Adv. Manuf. Technol..

[9] Henerichs M., Voß R., Kuster F., Wegener K. (2015). Machining of carbon fiber reinforced plastics: Influence of tool geometry and fiber orientation on the machining forces. CIRP J. Manuf. Sci. Technol..

[10] Davim J.P. (2009). Machining of Composite Materials.

[11] Wang X., Zhang L. (2003). An experimental investigation into the orthogonal cutting of unidirectional fibre reinforced plastics. Int. J. Mach. Tools Manuf..

[12] Kumar D., Gururaja S. (2020). Machining damage and surface integrity evaluation during milling of UD-CFRP laminates: Dry vs. cryogenic. Compos. Struct..

[13] Nguyen-Dinh N., Zitoune R., Bouvet C., Leroux S. (2019). Surface integrity while trimming of composite structures: X-ray tomography analysis. Compos. Struct..

[14] Oliveira T.L.L., Zitoune R., Ancelotti A.C., da Cunha S.S. (2020). Smart machining: Monitoring of CFRP milling using AE and IR. Compos. Struct..

[15] Zhu Z., Kang R., Huang J., Zhu Y., Sun X. (2023). Investigation on cutting damage mechanism of carbon fiber reinforced polymer based on macro/microscopic simulation. Int. J. Adv. Manuf. Technol..

[16] Han L., Zhang J., Liu Y., Sun T. (2021). Effect of fiber orientation on depth sensing intra-laminar failure of unidirectional CFRP under nano-scratching. Compos. Part B Eng..

[17] Mata F., Gaitonde V., Karnik S., Davim J.P. (2008). Influence of cutting conditions on machinability aspects of PEEK, PEEK CF 30 and PEEK GF 30 composites using PCD tools. J. Mech. Work. Technol..

[18] Xu J., Huang X., Davim J.P., Ji M., Chen M. (2020). On the machining behavior of carbon fiber reinforced polyimide and PEEK thermoplastic composites. Polym. Composite..

[19] Caggiano A. (2018). Machining of Fibre Reinforced Plastic Composite Materials. Materials.

[20] Wang H., Qin X., Li H. (2015). Machinability analysis on helical milling of carbon fiber reinforced polymer. J. Adv. Mech. Des. Syst. Manuf..

[21] Zhang J., Luo T., Ye Z., Deng C., Luo D., Tao G., Cao H. (2024). Investigation on fiber fracture mechanism and milling force model of CF/PEEK by ultrasonic milling. Tribol. Int..

[22] Lin Z., Jiao H., Zhou L., Zhang G., Huang P., Zhao Z., Liu H., Huang Y., Zhou J., Long Y. (2023). Investigation on mechanism for water-gas jet-assisted laser processing of carbon fiber-reinforced polymer. J. Reinf. Plast. Compos..

[23] Rao S., Sethi A., Das A.K., Mandal N., Kiran P., Ghosh R., Dixit A.R., Mandal A. (2017). Fiber laser cutting of CFRP composites and process optimization through response surface methodology. Mater. Manuf. Process..

[24] Qiao Y., Chen T., Ma H., Liu Y., Tang A., Xiong W., Deng L. (2023). Experimental study on the sidewall quality of femtosecond laser drilling CFRP. Compos. Part B Eng..

[25] Lee C.-M., Woo W.-S., Kim D.-H., Oh W.-J., Oh N.-S. (2016). Laser-assisted hybrid processes: A review. Int. J. Precis. Eng. Manuf..

[26] Tao N., Chen G., Cai S., Fang W., Xiao Z. (2023). Improving hole quality of thick CFRP laminates through a laser-mechanical compound drilling process. J. Mater. Res. Technol..

[27] Li Z., Zheng H., Lim G., Chu P., Li L. (2010). Study on UV laser machining quality of carbon fibre reinforced composites. Compos. Part A Appl. Sci. Manuf..

[28] Chen L., Li M., Yang X. (2022). The feasibility of fast slotting thick CFRP laminate using fiber laser-CNC milling cooperative machining technique. Opt. Laser Technol..

[29] Fujihara K., Huang Z.-M., Ramakrishna S., Hamada H. (2004). Influence of processing conditions on bending property of continuous carbon fiber reinforced PEEK composites. Compos. Sci. Technol..

[30] Kong X., Wang Y., Liu X., Dang Z., Wang M. (2024). Research on the removal mechanism and surface damage of laser assisted cutting of CFRP materials. J. Manuf. Process..

[31] Liu S., Sun Y., Du Y., Zhang Z., Wu X. (2023). Investigating the material removal mechanism and cutting performance in ultrasonic vibration-assisted milling of carbon fibre reinforced thermoplastic. Mater. Res. Express.

[32] Qin X., Wu X., Li H., Li S., Zhang S., Jin Y. (2022). Numerical and experimental investigation of orthogonal cutting of carbon fiber-reinforced polyetheretherketone (CF/PEEK). Int. J. Adv. Manuf. Technol..

[33] Garcia-Gonzalez D., Rusinek A., Jankowiak T., Arias A. (2015). Mechanical impact behavior of polyether–ether–ketone (PEEK). Compos. Struct..

[34] Bouteiller P., Bleyer J., Sab K. (2022). Continuum damage analysis of delamination in composite laminates using a stress-based layerwise plate model. Int. J. Solids Struct..

[35] Benzeggagh M.L., Kenane M. (1996). Measurement of mixed-mode delamination fracture toughness of unidirectional glass/epoxy composites with mixed-mode bending apparatus. Compos. Sci. Technol..

[36] Li H.N., Wang J.P., Wu C.Q., Zhao Y.J., Xu J., Liu X., Zhu W.Q. (2020). Damage behaviors of unidirectional CFRP in orthogonal cutting: A comparison between single- and multiple-pass strategies. Compos. Part B Eng..

[37] Li M., Chen L., Yang X. (2021). A feasibility study on high-power fiber laser cutting of thick CFRP laminates using single-pass strategy. Opt. Laser Technol..

[38] Weber R., Hafner M., Michalowski A., Graf T. (2011). Minimum Damage in CFRP Laser Processing. Phys. Procedia.

[39] Qi Z., Zhang K., Cheng H., Wang D., Meng Q. (2015). Microscopic mechanism based force prediction in orthogonal cutting of unidirectional CFRP. Int. J. Adv. Manuf. Technol..

[40] Kong X., Dang Z., Liu X., Wang M. (2023). A comparative evaluation of laser assisted drilling CFRP with improved machining mechanism. J. Mater. Process. Tech..

[41] Li H., Qin X., He G., Jin Y., Sun D., Price M. (2016). Investigation of chip formation and fracture toughness in orthogonal cutting of UD-CFRP. Int. J. Adv. Manuf. Technol..

[42] Wang F.-J., Zhang B.-Y., Zhao M., Cheng D., Wang Z.-G. (2019). Evolution laws of fiber-matrix interface cracks in machining of carbon fiber reinforced polymer. Int. J. Adv. Manuf. Technol..

[43] Zhu T., Ren Z., Xu J., Shen L., Xiao C., Zhang C., Zhou X., Jian X. (2023). Damage evolution model and failure mechanism of continuous carbon fiber-reinforced thermoplastic resin matrix composite materials. Compos. Sci. Technol..

[44] Wang C., Wen L., Ming W., An Q., Chen M. (2018). Experimental study on effects of fiber cutting angle in milling of high-strength unidirectional carbon fiber–reinforced polymer laminates. Proc. Inst. Mech. Eng. Part B J. Eng. Manuf..

[45] Gong S.L. (2016). Advanced Laser Processing Technology.

